# The role of music therapy and music-based interventions in male mental health: a narrative review of mechanisms, barriers, and clinical implications

**DOI:** 10.3389/fpubh.2026.1899615

**Published:** 2026-07-15

**Authors:** Jared Kyle Sieusahai

**Affiliations:** Faculty of Medicine and Dentistry, University of Alberta, Edmonton, AB, Canada

**Keywords:** emotion regulation, gateway mechanism, help-seeking, male mental health, masculinity norms, music therapy, PTSD, veterans

## Abstract

**Background/Objectives:**

Men bear a disproportionate burden of undertreated mental health problems globally yet consistently underutilize formal psychological services, primarily due to traditional masculine norms that frame help-seeking as incompatible with masculine identity. Music therapy and music-based interventions offer a promising pathway that may circumvent these barriers by providing masculinity-congruent, non-clinical contexts for emotional expression and social connection. This narrative review synthesizes evidence on the mechanisms, outcomes, and clinical implications of music therapy and music-based interventions for male mental health, advancing the gateway mechanism hypothesis as a novel theoretical framework.

**Methods:**

A systematic search was conducted across PubMed and the University of Alberta Library Primo discovery platform in June 2026, searching across PsycINFO, CINAHL, MEDLINE, Academic Search Complete, Web of Science, Scopus, and additional databases. Search strings targeted music therapy in combination with male mental health, depression, anxiety, veterans, PTSD, and related terms, yielding a combined multi-database pool of approximately 1,243 results. After screening for relevance and applying inclusion criteria, 38 peer-reviewed sources were included in this narrative review conducted in accordance with SANRA guidelines.

**Results:**

Evidence from randomized controlled trials, systematic reviews, and qualitative studies supports music therapy's effectiveness for depression, anxiety, PTSD, and trauma across predominantly male populations, with the strongest evidence in military veterans. The gateway mechanism hypothesis proposes that music functions as a masculinity-congruent, low-threat entry point through which men gradually develop psychological safety, engage in licensed emotional expression, and move toward help-seeking. The most direct experimental support comes from the Boys Do Cry RCT, which found that a music video significantly improved men's help-seeking intentions compared to non-musical control conditions. Evidence gaps persist around racialized, Indigenous, and 2SLGBTQ+ men, and around non-Western and low-income contexts. Effect sizes where reported are small to moderate; most studies are limited by small samples, heterogeneous designs, and absence of sex-disaggregated reporting.

**Conclusions:**

Music therapy represents a clinically meaningful, non-stigmatizing, and evidence-supported pathway to male mental health engagement. Practical implications include framing music-based programs around musical participation rather than mental health, prioritizing group formats, and selecting genres that align with participants' masculine cultural identity. Future research should employ longitudinal designs testing the gateway mechanism directly, incorporate validated masculinity norms instruments, ensure sex-disaggregated reporting, and develop culturally adapted approaches for diverse male populations globally.

## 1 Introduction

Men experience substantially elevated rates of suicide, substance use disorders, and untreated depression and anxiety relative to women, yet consistently underutilize formal mental health services across the life course (1–3). In Canada, men account for approximately 75% of suicide deaths (4), and only a minority of men over 12 report seeking professional help for emotional or mental health concerns (5). Similar patterns are documented across Australia, the United Kingdom, the United States, and other Western countries, reflecting a structural feature of how men relate to psychological help-seeking rather than a population-specific anomaly (6, 7).

The primary explanation for this pattern is well-established: traditional masculinity norms emphasizing self-reliance, emotional stoicism, and strength create a framework within which acknowledging psychological distress and seeking help from a professional are experienced as incompatible with masculine identity (6, 8). Connell's concept of hegemonic masculinity describes how dominant masculine ideals are reproduced through social practice and create structural barriers to health-seeking behavior (9). Addis and Mahalik's (10) framework of masculine role conflict further explains how men experience tension between their need for help and the perceived incompatibility of help-seeking with masculine self-conception. The result is a population that bears a substantial mental health burden but is systematically less likely than women to access the services designed to address it.

Music therapy and music-based interventions offer a theoretically compelling and empirically supported alternative pathway. Unlike formal psychological services, music-based engagement does not require men to identify as patients, acknowledge vulnerability, or seek help from an expert. Instead, it offers a masculinity-congruent entry point through shared activity, group participation, and creative engagement that may create the relational and emotional conditions through which men begin to open up, connect, and ultimately access support they would not otherwise seek.

Several reviews have examined music therapy's effectiveness for depression (11), anxiety (12), PTSD (13), and specific populations such as veterans (14). However, no existing review has specifically synthesized the evidence on music therapy as a pathway to male mental health engagement, integrating the masculinity and help-seeking literature with the music therapy evidence base. The specific contribution of this review is the formulation of the gateway mechanism hypothesis as a theoretical framework that explains why music-based interventions may be particularly effective for men, and the synthesis of converging empirical evidence that supports and qualifies this hypothesis. This review also explicitly distinguishes between credentialed music therapy delivered by trained therapists and broader music-based activities, a distinction that has important implications for interpreting the evidence and translating it into clinical practice.

This review is intentionally scoped to men as a population group because the help-seeking barriers, mechanisms of engagement, and clinical implications differ substantially between men and mixed-gender populations. This scoping does not imply that music therapy is more effective for men than for other populations, but rather that the specific interaction between masculine norms and music-based engagement merits dedicated theoretical and empirical attention.

## 2 Methods

This narrative review was conducted in accordance with SANRA guidelines [Baethge et al. (15)]. Narrative reviews synthesize evidence thematically and critically without the exhaustive systematic search processes of systematic reviews; they are appropriate when the goal is to develop a theoretical framework and critically examine an emerging evidence base rather than to produce a comprehensive inventory of all available evidence. The current review is explicitly presented as a narrative review throughout and employs narrative synthesis rather than meta-analytic pooling.

### 2.1 Search strategy

A systematic literature search was conducted across two platforms in June 2026. The University of Alberta Library Primo discovery platform was searched, aggregating across PsycINFO, CINAHL, MEDLINE, Academic Search Complete, Web of Science, Scopus, and additional databases. Five search term pairs were applied: music therapy AND male mental health (1,075 results); music therapy AND masculinity help-seeking (2 results); music therapy AND veterans PTSD (77 results); music therapy AND depression anxiety men (78 results); and music AND help-seeking men mental health (11 results), yielding a combined multi-database pool of approximately 1,243 results before deduplication. PubMed was additionally searched using seven primary search strings: “music therapy” [Title/Abstract] AND “depression” [Title/Abstract] AND “men” [Title/Abstract] (12 results); “music therapy” [Title/Abstract] AND “anxiety” [Title/Abstract] AND “men” [Title/Abstract] (21 results); “music therapy” [Title/Abstract] AND “mental health” [Title/Abstract] AND “men” [Title/Abstract] (4 results); “music therapy” [Title/Abstract] AND “masculinity” [Title/Abstract] (0 results); “music therapy” [Title/Abstract] AND “veterans” [Title/Abstract] (20 results); “music therapy” [Title/Abstract] AND “older men” [Title/Abstract] (1 result); and “music therapy” [Title/Abstract] AND “social isolation” [Title/Abstract] AND “men” [Title/Abstract] (1 result), yielding a combined deduplicated PubMed total of 44 results. Supplementary PubMed searches targeted emotion regulation (27), social connection (9), help-seeking (21), PTSD (31), trauma and men (1), community and men (1), and wellbeing and male (3), yielding a total deduplicated PubMed pool of 138 results. Reference lists of all included systematic reviews were hand-searched to identify additional relevant literature.

The zero-result finding for “music therapy AND masculinity” in PubMed is itself a significant finding documented in this review: there is no published literature that directly connects music therapy practice with the theoretical framework of masculinity norms. This gap provides the primary justification for this review's theoretical contribution.

### 2.2 Inclusion and exclusion criteria

Studies were included if they: (1) examined music therapy or music-based interventions as a primary or significant component of a mental health intervention; (2) addressed outcomes relevant to male mental health including depression, anxiety, PTSD, trauma, social isolation, emotional regulation, or help-seeking; (3) involved male or predominantly male populations, or provided sex-disaggregated data enabling male-specific conclusions; (4) were published in English between 2000 and 2026; and (5) were peer-reviewed. Contextual literature on masculinity norms, male help-seeking barriers, and music psychology was included where it directly informed the mechanistic framework. Studies were excluded if they focused exclusively on female populations, pediatric populations under 16, or if music was an incidental rather than primary component. The English-language restriction is acknowledged as a limitation that may have excluded relevant non-Western literature.

### 2.3 Screening and study selection

Titles and abstracts from both search platforms were screened for relevance against the inclusion and exclusion criteria by the author. Full texts of relevant studies were retrieved and assessed. A total of 38 peer-reviewed sources were included. The screening process involved three stages: title and abstract screening of all results from both platforms; full-text review of studies passing initial screening; and hand-searching of reference lists of included systematic reviews. Studies identified through hand-searching were assessed against the same inclusion criteria. Given the absence of any existing literature directly connecting music therapy and masculinity norms, the review necessarily draws on both direct evidence from music therapy trials and contextual evidence from masculinity and help-seeking research to build the gateway mechanism framework.

### 2.4 Quality assessment

Methodological quality is assessed descriptively throughout the Results sections and summarized in Table 1 using a four-level rating: high (Cochrane reviews, multicenter RCTs, large systematic reviews with comprehensive search strategies); moderate (RCTs or systematic reviews with identified limitations, mixed-methods studies with clear methodology, foundational theoretical frameworks); low-moderate (pilot studies, single-site qualitative studies, program evaluations with limited methodological detail); and low (case studies, commentaries, perspective pieces, historical reviews, theoretical papers). This approach is consistent with narrative review standards as described by Grant and Booth (16). The distinction between direct and indirect evidence for the gateway mechanism hypothesis is made explicit throughout the Results sections.

**Table 1:** Summary of included studies with quality assessment (NR = not reported).

References	Country	Design	Sample	Population	Intervention	Key findings	Quality
Bilsker et al. (1)	Canada	Narrative review	N/A	Canadian men	N/A	High suicide rates, underutilization of MH services, risky coping; masculine norms as primary driver.	Moderate
Seidler et al. (6)	International	Systematic review	Multiple studies	Men with depression	N/A	Masculine norm conformity consistently associated with lower help-seeking willingness.	High
Mokhwelepa and Sumbane (3)	South Africa	Quantitative	NR	Men	N/A	Traditional masculinity norms significantly predicted lower willingness to seek MH support.	Moderate
Connell (9)	International	Theoretical	N/A	General	N/A	Hegemonic masculinity framework: dominant masculine ideals reproduced through social practice creating health-seeking barriers.	Foundational
Addis and Mahalik (10)	USA	Theoretical/review	N/A	Men	N/A	Masculine role conflict framework: help-seeking perceived as incompatible with masculine self-conception.	Foundational
Sharp et al. (17)	Canada	Photovoice	*n* = 65	Canadian men	N/A	Dual paradoxes: wanting connection while unable to seek it; masculine norms produce concealment.	Moderate
Sharp et al. (7)	Canada	Survey	NR	Employed Canadian men	N/A	Over half kept feelings to themselves; almost half preferred not discussing problems with others.	Moderate
Nicholas et al. (21)	Australia	RCT	NR	Adult men	Music video intervention	Music video significantly improved help-seeking intentions and attitudes vs control condition.	High
Nicholas et al. (22)	Australia	Qualitative	NR	Australian men	Music video intervention	Men responded positively to help-seeking framed as strength; musical format reduced stigma vs text.	Moderate
Adeyola and Allen (23)	USA	Qualitative	NR	Black men	Rap music therapy	Rap format created culturally congruent context for emotional expression not possible in conventional therapy.	Low-Moderate
Roth (24)	International	Mixed methods	NR	General	Group improvisation	Co-improvisation produced mutual emotional engagement and interpersonal trust enabling disclosure.	Moderate
Huang and Chen (25)	China	Quasi-experimental	NR	Male inpatients	Group MT emotion regulation	Significant reductions in depression and anxiety relative to treatment as usual.	Moderate
Aalbers et al. (11)	International	Cochrane review	Multiple RCTs	Depression	Music therapy	Significant reductions in depressive symptoms; effect sizes comparable to brief psychological interventions.	High
Gaebel et al. (26)	Germany	RCT substudy	NR	Depressive women	Group MT	Significant cortisol and emotion regulation improvements; biological evidence for stress-reduction pathway.	Moderate
Ma et al. (13)	International	Meta-analysis	Multiple RCTs	PTSD	Music therapy	Significant pooled effect on PTSD symptom severity; heterogeneity substantial; bias high.	Moderate
Wang et al. (27)	International	Systematic review	Multiple studies	PTSD	Music interventions	18 studies showing PTSD symptom improvements; veteran population showed most robust effects.	Moderate
Uomoto et al. (14)	USA	Systematic review	Multiple studies	Veterans, TBI/PTSD	Multiple MT modalities	Consistent evidence across receptive, improvisation, songwriting for emotional regulation and social reintegration.	Moderate-high
Story et al. (28)	USA	Proof-of-concept RCT	NR	Veterans with pain	Telehealth music imagery	Significant pain outcome improvements; telehealth delivery feasible.	Moderate
Story et al. (29)	USA	Feasibility study	NR	Veterans, PTSD	Music imagery/listening	High engagement rates contrasting with typical dropout from conventional PTSD treatments.	Low-moderate
Story et al. (44)	USA	Qualitative	NR	Veterans with chronic pain	Music	Music provided emotional regulation, social connection, sense of control beyond formal treatment.	Low-moderate
Vaudreuil et al. (45)	USA	Mixed methods	NR	Military veterans	Telehealth MT	MT maintained benefits for veterans via telehealth; in-person preferred for group formats.	Low-Moderate
Drozd (35)	USA	Perspective	N/A	Military culture	MT	Military culture features make MT particularly well-suited: shared identity, non-clinical framing.	Low
Reschke-Hernandez (46)	USA	Historical review	N/A	WWI troops	Historical MT	Music therapy with shell shock patients in WWI; historical precedent for MT with male military populations.	Low
Powers et al. (32)	USA	Program evaluation	NR	Homeless veterans	Group MT	MT promoted movement from isolation to community; new social relationships through musical participation.	Low
Gooding et al. (33)	USA	Program evaluation	NR	Veterans	Community MT program	Sustained engagement; rock/country/folk/blues preferred; singing and instrument play most common.	Low-moderate
Beck et al. (36)	Denmark	RCT	NR	Traumatized refugees	MT vs standard treatment	MT non-inferior to verbal standard treatment; higher engagement in male participants.	Moderate
Beck et al. (37)	Denmark	Feasibility study	NR	Traumatized refugees	MT	MT acceptable and beneficial; non-verbal and musical aspects preferred by male participants.	Low-moderate
Carr et al. (38)	UK	Quasi-experimental	NR	PTSD patients	Group MT	Improvements in PTSD severity and social functioning; group peer support as key mechanism.	Moderate
Baker et al. (31)	International	Systematic review	Multiple studies	PTSD	Creative arts therapies	Promising but inconsistent evidence; some studies showed no significant effects on primary PTSD outcomes.	Moderate
Landis-Shack et al. (30)	USA	Theoretical review	N/A	PTSD	MT	Compelling theoretical rationale; empirical base limited by small samples and heterogeneous designs.	Low-moderate
Haddad et al. (34)	USA	Pilot RCT	NR	Lonely older adults	Remote personalized MT	Significant improvements in loneliness and wellbeing; male participants showed strong engagement.	Low-moderate
Wilson and Cordier (18)	International	Narrative review	Multiple studies	Men's Shed members	Men's Shed participation	Reduced social isolation, improved wellbeing through activity-based engagement; health by stealth mechanism.	Moderate
Cordier and Wilson (19)	International	Review	Multiple studies	Men's Shed members	Men's Shed participation	Community-based Men's Sheds promote male health through masculinity-congruent social engagement.	Moderate
Foettinger et al. (39)	International	Mixed-methods SR	52 studies	Men's Shed members	Men's Shed participation	Consistent association with reduced isolation and improved wellbeing; health by stealth confirmed.	Moderate-high
McEvoy et al. (20)	Australia	Survey	*n* = 333	Men's Shed members	Men's Shed participation	Psychological safety predicted engagement; engagement predicted social networks, meaning, wellbeing.	Moderate
Cavanagh et al. (41)	Australia	Qualitative	NR	Indigenous men	Culturally adapted MT/activities	Indigenous men thriving through culturally appropriate community activities; elder-led programs key.	Low-moderate
Koelsch and Bradt (43)	International	Narrative review	N/A	General	Music neuroscience	Music engages reward, social, emotion-regulation circuits; biological grounding for MT mechanisms.	Moderate
Mahalik et al. (42)	USA	Scale development	NR	Men	CMNI-46	CMNI-46 development; validated instrument for measuring conformity to masculine norms.	High
Alexandre et al. (40)	DRC	Mixed methods	NR	Conflict-zone patients	MT	Gender-differentiated responses to MT in DRC; culturally grounded MT approaches required in non-Western contexts.	Low-moderate

### 2.5 Distinction between music therapy and music-based activities

A clarification of terminology is essential before presenting findings. Credentialed music therapy refers to interventions delivered by trained and certified music therapists (e.g., MT-BC in the United States, MTA in Canada) who conduct clinical assessment, develop individualized treatment plans, and apply music therapeutically within a therapeutic relationship. Music medicine refers to the use of music as a medical adjunct, typically pre-recorded music, without therapist presence. Music-based activities refers to any organized engagement with music in community, educational, or recreational contexts without formal therapeutic structure, including Men's Sheds programs incorporating musical activities, community music groups, and online music communities. This review includes evidence from all three categories where relevant, but distinguishes between them throughout. Evidence from music-based activities and community music programs is presented as contextual support for the gateway mechanism rather than as evidence for music therapy specifically.

## 3 Results

Table 1 presents a summary of all 38 included studies with quality ratings.

### 3.1 Male mental health burden and help-seeking barriers

Men's underutilization of formal mental health services is one of the most consistently documented patterns in mental health epidemiology. Systematic reviews of masculinity norms and help-seeking consistently report that traditional masculine ideals of strength, self-reliance, and emotional control are the primary drivers of this pattern, operating through self-stigma, fear of appearing weak, low mental health literacy, and preference for informal or self-reliant coping strategies (6, 8, 15). Seidler et al.'s (6) high-quality systematic review of masculinity and help-seeking for depression found that conformity to masculine norms was consistently associated with lower willingness to seek professional help and greater endorsement of self-reliant coping across multiple cultures and age groups. Mokhwelepa and Sumbane's (8) quantitative study from South Africa extends this finding beyond Western contexts, documenting that traditional masculinity norms significantly predicted lower willingness to seek mental health support in a non-Western male sample.

Sharp et al.'s (17) photovoice study of 65 Canadian men identified dual paradoxes that structure men's relationship to peer support: simultaneously wanting to support others while being deeply reluctant to seek support themselves, and valuing emotional authenticity while suppressing it in practice. These paradoxes are not individual psychological failures but structural features of how masculine identity interacts with help-seeking contexts explicitly framed around mental health and emotional disclosure. A survey of Canadian men in the workplace (7) found that over half reported keeping feelings to themselves and almost half preferred not discussing problems with others. These findings are consistent across Western national contexts but evidence from non-Western, low-income, and culturally diverse male populations remains limited.

The quality of evidence on masculine norms and help-seeking is moderate to high for cross-sectional relationships, but most evidence is cross-sectional and therefore cannot support causal conclusions about whether changing masculine norms produces changes in help-seeking behavior. Longitudinal and intervention-focused research remains sparse.

### 3.2 The gateway mechanism hypothesis

#### 3.2.1 Theoretical framework

The gateway mechanism hypothesis proposes that music therapy and music-based interventions may be effective entry points for men's mental health support because they provide a masculinity-congruent context that may circumvent the help-seeking barriers described above. This hypothesis is explicitly presented as a theoretical framework supported by converging indirect evidence rather than a confirmed empirical finding. It draws on and extends related concepts including health by stealth (18, 19), gender-sensitive engagement (8), and activity-based approaches to men's help-seeking (20), while specifying music as an activity with particular gateway properties and proposing a sequential psychological process through which engagement produces conditions for help-seeking.

The gateway mechanism is distinguished from health by stealth by its specificity about the psychological process through which activity-based engagement produces health outcomes. It is distinguished from gender-sensitive engagement by its focus on the entry point to engagement rather than on adapting existing services. It proposes four sequential stages: initial engagement through the intrinsic appeal of musical activity, without requiring acknowledgment of mental health need; development of psychological safety through musical synchrony and shared activity; emotional expression through and about music, licensed by cultural norms around musical emotional response; and eventual disclosure and help-seeking that would not have occurred without the musical entry point.

#### 3.2.2 Evidence consistent with the gateway mechanism

The most direct experimental support for the gateway mechanism comes from the Boys Do Cry randomized controlled trial (21), which found that exposure to a music video specifically designed to encourage men to seek help produced significant improvements in help-seeking intentions and attitudes compared to a control condition. This is the only study identified in this review that directly and experimentally tests a prediction of the gateway hypothesis—specifically that music-based content can shift the masculine norms that prevent men from seeking support. A qualitative companion study (22) found that the musical format made the mental health message more accessible and less stigmatizing than non-musical equivalents, providing a mechanism-consistent explanation for the trial's main findings.

Adeyola and Allen's (23) qualitative study of Black men's engagement with rap music therapy found that the rap format created a culturally congruent context within which Black men expressed anger, depression, and vulnerability they would not articulate in conventional therapeutic settings. This finding is consistent with the gateway mechanism's prediction that cultural specificity in musical format determines gateway effectiveness, though it was not designed to test the hypothesis directly. Roth's (24) study of shared flow through group improvisation documented states of mutual emotional engagement and interpersonal trust that participants described as enabling conversations not otherwise possible, consistent with the gateway mechanism's second and third stages, though again not designed as a direct test.

It is important to be explicit about what this evidence does and does not support. The Boys Do Cry RCT (21) provides direct experimental evidence that music-based content can shift help-seeking intentions. The remaining evidence is consistent with gateway mechanism predictions but does not directly test them. No published study has tracked men through the sequential stages of the gateway mechanism from initial musical engagement through to formal help-seeking, which is what direct empirical confirmation of the hypothesis would require. The framework is therefore best understood as a theoretically grounded and empirically supported hypothesis requiring prospective longitudinal testing.

### 3.3 Music therapy outcomes for male mental health

#### 3.3.1 Depression and anxiety

Aalbers et al.'s (11) cochrane review of music therapy for depression found significant reductions in depressive symptoms across multiple study designs, rated High quality, with effect sizes comparable to brief psychological interventions. However, this review did not report sex-disaggregated findings, limiting conclusions about male-specific effects. Huang and Chen's (25) quasi-experimental study of group music therapy based on emotion-regulation skills specifically in male inpatients found significant reductions in depression and anxiety relative to treatment as usual, providing the most direct evidence for depression and anxiety outcomes in a male-specific sample. Effect sizes were not reported, limiting quantitative synthesis.

Null and mixed findings deserve explicit acknowledgment. Gaebel et al.'s (26) RCT substudy, conducted with depressive women, documented significant cortisol and emotion regulation improvements, providing biological evidence for stress-reduction mechanisms that may generalize to male populations but has not been tested in men specifically. The absence of male-specific depression and anxiety RCTs represents a significant gap: the evidence base for these outcomes in men is primarily extrapolated from mixed-gender studies rather than established in male-specific designs.

#### 3.3.2 PTSD and trauma

The strongest and most consistent evidence for music therapy in male mental health comes from the PTSD and trauma literature, where military veterans represent the primary population studied. Ma et al.'s (13) meta-analysis found a significant pooled effect on PTSD symptom severity across music therapy studies, though heterogeneity was substantial and risk of bias was high in most included trials. Wang et al.'s (27) systematic review identified 18 studies demonstrating improvements in PTSD symptoms, emotional regulation, and quality of life, with veteran populations showing the most robust effects. Uomoto et al.'s (14) moderate-high quality systematic review documented consistent evidence across receptive listening, improvisation, and songwriting modalities for emotional regulation and social reintegration outcomes in veterans.

Several RCTs specifically in veteran populations provide stronger causal evidence. Story et al. (28, 29) conducted a proof-of-concept RCT of telehealth music imagery for pain in veterans, finding significant improvements and demonstrating telehealth delivery feasibility. High engagement rates in these veteran music therapy studies contrast with typical dropout from conventional PTSD treatments, providing naturalistic evidence that music therapy reaches men who would not otherwise engage with services—a pattern consistent with the gateway mechanism.

Landis-Shack et al.'s (30) theoretical review noted that while the theoretical rationale for music therapy in PTSD is compelling, the empirical evidence base remains limited by small samples, heterogeneous interventions, and high risk of bias. Baker et al.'s (31) systematic review of creative arts therapies for PTSD found promising but inconsistent evidence, with some studies showing no significant effects on primary outcomes. These null and mixed findings are important to acknowledge and indicate that the field cannot yet confidently position music therapy as a first-line treatment for PTSD, even in veteran populations.

#### 3.3.3 Social isolation and connection

Powers et al.'s (32) program evaluation documented music therapy promoting movement from isolation to community in homeless veterans, with participants reporting new social relationships formed through musical participation. Gooding et al.'s (33) programmatic evaluation found sustained engagement over 12 months in a community music program for veterans in supported housing, with rock, country, blues, and folk music preferred and singing and instrument play as primary modalities—a pattern consistent with masculinity-congruent musical formats producing engagement. Haddad et al.'s (34) pilot RCT of remotely delivered music therapy for lonely older adults found significant loneliness and wellbeing improvements, with male participants showing strong engagement. Evidence quality in this domain is predominantly low to low-moderate.

### 3.4 Specific male populations

#### 3.4.1 Military veterans

Military veterans represent the most extensively studied male population in the music therapy literature and provide the strongest evidence base for music therapy's effectiveness in a predominantly male context. The Veterans Health Administration has developed and evaluated music therapy programs across multiple facilities (14, 28, 29, 32). Drozd (35) identifies features of military culture—shared identity, preference for non-clinical framing, and the historical resonance of music in military contexts—that make music therapy particularly well-suited to this population. The quality of veteran music therapy evidence is moderate overall. The strongest studies are the Story et al. RCT series (28, 29) and the Uomoto et al. (14) systematic review.

#### 3.4.2 Non-military PTSD and trauma

Beck et al.'s (36) RCT found that music therapy was non-inferior to verbal standard treatment for traumatized refugees, with higher engagement among male participants who reported greater comfort with non-verbal and musical aspects. A companion feasibility study (37) confirmed acceptability and benefit for this population. Carr et al.'s (38) quasi-experimental study of group music therapy for persistent PTSD documented improvements in PTSD severity and social functioning. The finding that music therapy is non-inferior to verbal treatment for traumatized populations including men who resist verbal formats is clinically significant, though effect sizes were not reported and sample sizes were small.

#### 3.4.3 Older and retired men

Haddad et al.'s (34) pilot RCT provides the most direct evidence for music therapy with older men. The Men's Sheds literature (18–20, 39) provides broader evidence for the health by stealth model in this population, with mixed-methods systematic reviews consistently documenting reduced isolation and improved wellbeing through activity-based engagement. McEvoy et al.'s (39) path-model survey of 333 Shed members documented a sequential structure—psychological safety predicting engagement, engagement predicting social networks and meaning, both predicting wellbeing—that mirrors the gateway mechanism's stages and suggests the framework may generalize beyond music to other activity-based interventions for older men.

### 3.5 Race, culture, and equity

The music therapy literature for male mental health reflects significant demographic narrowness. Most studies involve white, Western, English-speaking men, with limited evidence for the specific experiences of racialized, Indigenous, immigrant, and sexually diverse male populations.

Adeyola and Allen (23) provide the most direct evidence for culturally specific music therapy with racialized men, finding that rap music therapy created a congruent context for Black men's emotional expression that conventional therapy had not achieved. Alexandre et al. (40) documented gender-differentiated responses to music therapy in the Democratic Republic of Congo, finding that culturally grounded approaches were prerequisites for therapeutic engagement in a non-Western conflict context. These findings collectively suggest that the musical format's cultural resonance with masculine identity is not a minor implementation detail but a determinant of gateway effectiveness—and that this cultural specificity must be developed for each population rather than assumed from Western evidence.

For Indigenous men in Canada and Australia, Cavanagh et al. (41) documented the importance of culturally adapted, elder-led community programming that centers Indigenous masculine identities and community governance. No published studies specifically examine music therapy outcomes for Indigenous men. For 2SLGBTQ+ men, no included studies addressed music therapy outcomes specifically, representing a particularly significant gap given elevated mental health burden and barriers to care in this population. Future research must center these populations through community-based participatory approaches rather than adapting Western protocols.

The absence of non-Western evidence is also partly a function of this review's English-language restriction. Relevant literature may exist in Chinese, Korean, Japanese, Portuguese, Arabic, and other languages that was not captured by the search strategy. This limitation must be acknowledged as constraining the global generalizability of the gateway mechanism framework as currently formulated.

### 3.6 Music therapy and formal mental health services

A concern about non-clinical approaches to male mental health is that they may function as substitutes for rather than gateways to formal services. The available evidence does not support this concern. Studies of music therapy in clinical contexts consistently position music-based interventions as complements to rather than replacements for formal psychological treatment (14, 30, 31). The Boys Do Cry RCT (21) specifically found that music video exposure increased help-seeking intentions rather than providing an alternative to help-seeking, directly supporting the gateway rather than substitute interpretation. Music therapists working in clinical settings should explicitly position their services as front-door interventions that increase engagement with the broader mental health system.

### 3.7 Reporting quality and evidence gaps

The male mental health music therapy evidence base has significant methodological limitations that must temper clinical claims. Most studies are small, use varied outcome measures, and few are specifically designed to examine male populations as a distinct group. The most fundamental problem is that sex-disaggregated data are rarely reported: many music therapy studies include both male and female participants but do not report outcomes separately by sex, making it impossible to determine whether effects differ between men and women and what the specific evidence base for male populations actually is (11, 13, 27).

Publication bias is a likely concern in this literature: studies with positive findings are more likely to be published, and the consistently positive tone of the veteran music therapy literature may not fully represent null or negative findings that were not submitted or accepted for publication. The heterogeneity of interventions, settings, and outcome measures across studies prevents meta-analytic pooling and makes cross-study comparison unreliable. Effect sizes, where reported, are small to moderate, consistent with other brief psychosocial interventions but not large enough to support strong standalone clinical claims.

The zero-result finding for ‘music therapy AND masculinity' in PubMed remains the most significant single finding of this review's search strategy. No published literature directly connects music therapy practice with the theoretical framework that most powerfully explains its potential effectiveness for men. Validated instruments for masculinity norms measurement such as the CMNI-46 (42) have not been applied in music therapy research, meaning that the gateway mechanism's central theoretical claim—that music therapy works for men because it is masculinity-congruent—remains a well-supported hypothesis rather than an empirically confirmed mechanism.

## 4 Discussion

This narrative review synthesizes evidence on music therapy and music-based interventions for male mental health, advancing the gateway mechanism hypothesis as a theoretical framework for understanding how music may reach men who resist conventional mental health services. The central finding is that converging evidence from veteran music therapy programs, the Boys Do Cry RCT, the Men's Sheds literature, and culturally specific music therapy research is consistent with the gateway mechanism's predictions, while no study has directly tested the hypothesis through a longitudinal design tracking men from initial musical engagement through to formal help-seeking.

The gateway mechanism extends existing concepts of health by stealth (18, 19) and gender-sensitive engagement (8) by specifying the sequential psychological process through which activity-based musical engagement produces conditions for help-seeking, and by identifying music's specific neurobiological (43) and cultural properties as determinants of its gateway effectiveness. This theoretical contribution is novel in the sense that no existing review has formally synthesized the masculinity and music therapy literatures, but the hypothesis itself draws on and extends well-established frameworks rather than emerging from nothing.

The evidence is most robust for PTSD and trauma outcomes in veteran populations, where multiple systematic reviews and several RCTs document music therapy's effectiveness in a population with pronounced masculine norms and documented resistance to conventional services. The consistent finding that this population engages with music therapy at higher rates than with conventional services is itself theoretically significant, even where specific causal mechanisms have not been isolated. Evidence for depression and anxiety in non-veteran male populations is weaker, primarily because male-specific designs are rare and sex-disaggregated reporting is almost entirely absent from the broader music therapy literature.

The equity gaps in the literature are substantial. The gateway mechanism has been developed almost entirely from evidence with white, Western, cisgender, heterosexual men, and its applicability to men whose masculine identities are shaped by different cultural frameworks has not been tested. Adeyola and Allen's (23) finding that rap music therapy's cultural specificity was a prerequisite for Black men's emotional engagement suggests that culturally adapted music therapy models are not merely desirable but clinically necessary for diverse male populations. This conclusion should inform both research priorities and practice development.

### 4.1 Positionality and limitations

The author has volunteered as a live musician at the University of Alberta Hospital in Edmonton, Alberta, Canada through the Artists on the Wards program for 6 years, and manages an online guitar education community of approximately 30,000 members. These contexts provide direct observational familiarity with how music functions as a gateway to emotional expression and connection for men in both clinical and community settings. In the online community, men who engage initially around technical musical questions have progressively shared personal struggles as relational safety has developed over time. In the hospital context, male patients who would not initiate conversation with clinical staff have approached during musical performances to share their experiences. These observations are presented here as contextual background that informed the development of the gateway mechanism framework—they are not presented as empirical evidence and should not be interpreted as research data.

The author's personal investment in music's value for mental health represents a potential source of confirmation bias in evidence selection, interpretation, and framing. To mitigate this, the review explicitly acknowledges null and mixed findings, uses cautious language about causal claims, distinguishes between direct and indirect evidence for the gateway mechanism, and restricts all empirical claims to what the peer-reviewed literature supports. Readers should weigh the positionality disclosure accordingly.

The primary limitations of this review are: restriction to English-language literature, which may have excluded relevant non-Western evidence; the absence of sex-disaggregated reporting in most included studies, which limits male-specific conclusions; the predominance of low to moderate quality evidence; the absence of any published study directly testing the gateway mechanism through a longitudinal design; and the concentration of evidence in white, Western, veteran populations that limits the review's global applicability.

## 5 Conclusion

Music therapy and music-based interventions represent a clinically meaningful, non-stigmatizing, and evidence-supported pathway to male mental health engagement that has been substantially underutilized in both research and clinical practice. The gateway mechanism hypothesis proposed here—whereby music functions as a masculinity-congruent, low-threat entry point creating conditions for gradual emotional opening and eventual help-seeking—provides a coherent theoretical account of why music-based approaches may reach men who resist all other forms of mental health support, and has direct implications for clinical program design.

For clinicians and service providers, the practical implications are as follows. Music-based programs for male mental health should be framed around musical participation rather than mental health support, using the music itself as the primary stated purpose. Genre and modality should be selected to maximize alignment with participants' masculine cultural identity. Group formats that create conditions for musical synchrony and peer social connection should be prioritized. Programs should be explicitly positioned as entry points to the broader mental health system rather than as endpoints, with referral pathways to formal services built into program design.

For policymakers and health system planners, the evidence supports investment in community music programs as front-door interventions for male mental health, particularly in settings where formal music therapy staffing is not available. Men's Sheds programs incorporating musical activity, veteran community music programs, and online music communities represent scalable models that align with the gateway mechanism framework and have documented engagement in populations with high masculine norm conformity.

Future research should: employ longitudinal designs that track men from initial music-based engagement through to formal help-seeking over time, to directly test the gateway mechanism's sequential predictions; incorporate validated masculinity norms instruments including the CMNI-46 (42) alongside mental health outcome measures; ensure sex-disaggregated reporting in all music therapy trials; develop and evaluate culturally adapted music therapy models for Black, Indigenous, racialized, immigrant, and 2SLGBTQ+ men through community-based participatory research; conduct multilingual searches in future reviews to capture non-Western literature; and undertake direct comparative trials of music-based entry point programs vs. standard mental health outreach for men to establish the incremental value of the gateway approach.
